# FGO-PMB: A Factor Graph Optimized Poisson Multi-Bernoulli Filter for Accurate Online 3D Multi-Object Tracking

**DOI:** 10.3390/s26020591

**Published:** 2026-01-15

**Authors:** Jingyi Jin, Jindong Zhang, Yiming Wang, Yitong Liu

**Affiliations:** 1 College of Computer Science and Technology, Jilin University, Changchun 130012, China; 2 Key Laboratory of Symbolic Computation and Knowledge Engineering of Ministry of Education, Jilin University, Changchun 130012, China

**Keywords:** autonomous driving, lidar point cloud, 3D multi-object tracking, factor graph optimization, random finite set

## Abstract

Three-dimensional multi-object tracking (3D MOT) plays a vital role in enabling reliable perception for LiDAR-based autonomous systems. However, LiDAR measurements often exhibit sparsity, occlusion, and sensor noise that lead to uncertainty and instability in downstream tracking. To address these challenges, we propose FGO-PMB, a unified probabilistic framework that integrates the Poisson Multi-Bernoulli (PMB) filter from Random Finite Set (RFS) theory with Factor Graph Optimization (FGO) for robust LiDAR-based object tracking. In the proposed framework, object states, existence probabilities, and association weights are jointly formulated as optimizable variables within a factor graph. Four factors, including state transition, observation, existence, and association consistency, are formulated to uniformly encode the spatio-temporal constraints among these variables. By unifying the uncertainty modeling capability of RFS with the global optimization strength of FGO, the proposed framework achieves temporally consistent and uncertainty-aware estimation across continuous LiDAR scans. Experiments on KITTI and nuScenes indicate that the proposed method achieves competitive 3D MOT accuracy while maintaining real-time performance.

## 1. Introduction

In recent years, with the widespread application of LiDAR, 3D multi-object tracking (3D MOT) has received considerable attention in fields such as autonomous driving. LiDAR provides high-precision 3D point cloud data, which serves as a fundamental perception source for MOT, a core component of autonomous driving and computer vision. Three-dimensional MOT aims to track multiple dynamic objects by estimating their states and maintaining consistent identities over time. Specifically, in road traffic scenarios, 3D MOT is intended to accurately acquire the 3D positions, velocities, and other attributes of traffic participants using point cloud data, while ensuring consistent identity association across frames. With the advancement of autonomous driving technology, LiDAR-based 3D MOT plays a crucial role in achieving key functions such as real-time environmental perception, trajectory planning, and collision avoidance.

Despite the importance of 3D MOT, achieving accurate and robust tracking with LiDAR point cloud data in complex dynamic environments remains challenging. These challenges stem from a variety of sources of uncertainty, including uncertainty in the number of objects due to their birth and death, observation uncertainty resulting from sensor noise, missed detections, and false detections, and uncertainty in data association arising from proximity, occlusions, and similar appearances between objects. To address these challenges, traditional filtering and association methods [1,2,3,4] have been widely adopted in industry recently. However, their rigid association decisions and heuristic lifecycle management mechanisms make them less robust in the face of complex uncertainty. In recent years, methods using probabilistic graphical models [5,6] have improved tracking performance by incorporating richer contextual information. However, these methods often rely on heuristic cost functions rather than a unified probabilistic framework to model the uncertainty of object existence and data association [3,5]. Therefore, the development of a comprehensive and efficient unified framework to address this range of uncertainties remains a critical challenge in the field of 3D MOT [7,8,9].

Random Finite Sets (RFS) is a probabilistic model that can efficiently handle uncertainty and multi-object dynamics, making it particularly well-suited for MOT [10,11,12]. In the RFS framework, objects in the scene are no longer regarded as a set of independent random vectors but as an overall random set, whose number of elements and states are random variables. Since RFS uses a unified probabilistic representation, object birth, death, missed detections, false detections, and data association are naturally handled within a unified Bayesian filtering framework. The reliance of traditional methods on heuristic rules and hard decisions is thus eliminated. Especially advanced RFS filters, such as Poisson Multi-Bernoulli (PMB) [13] and its hybrids, provide explicit presence probability and state estimations for each potential object, resulting in high modeling capacity and robust tracking performance. However, typical RFS methods are limited by their online, recursive, frame-by-frame processing. This mechanism lacks the ability to consistently model and jointly process information over multiple time steps. This limitation makes it prone to local optima and fragmented trajectories when confronted with occlusions and other scenarios requiring global context.

Factor graph optimization (FGO) provides a probabilistic reasoning framework. It transforms the MOT problem into a global optimal estimation task by formulating the complex probabilistic reasoning problem as a graph consisting of variable nodes and factor nodes. FGO has been widely applied in fields such as simultaneous localization and mapping (SLAM), and it has been gradually extended to the cross-frame data association in multi-object scenarios [14,15]. In the factor graph framework, object states and associations are modeled as variable nodes, and constraints such as motion and observation are represented as factors. Global probabilistic dependencies among variables are established within the graph. By globally optimizing across temporal frames and spatial associations, all variables are jointly reasoned about in the time series, overcoming the limitations of frame-by-frame processing. However, global reasoning incurs significant computational overhead, limiting its applicability in real-time scenarios. As a result, a fixed-length sliding time window mechanism is commonly adopted to reduce complexity while retaining recent information. A more fundamental challenge lies in the fact that in FGO, the construction of factors often relies on heuristic cost functions, lacking a probabilistic modeling framework that can uniformly handle multiple uncertainties.

Thus, RFS and FGO show significant complementarity in MOT. In concrete terms, RFS provides a unified probabilistic modeling framework for dealing with uncertainty in object number and state, while FGO provides an optimization mechanism for global reasoning across time steps. Therefore, in this work, we propose an innovative framework that integrates RFS and FGO, where RFS serves as the probabilistic foundation for factor construction, allowing a tracker with both probabilistic completeness and global optimization capability.

To summarize, the main contributions are as follows:

- A novel 3D MOT framework named FGO-PMB is proposed that tightly integrates RFS with FGO. The object states, existence probabilities, and association weights in RFS are uniformly modeled as variable nodes in the factor graph. The factor graph models the probabilistic relationship among these uncertainties, enabling unified reasoning over state estimation, data association, and object lifecycle management.
- A set of probabilistic factors, including state transition, observation, existence, and association consistency, is designed to convert PMB priors into global optimization constraints. In particular, the existence factor models object birth and death as optimizable variables, and the association consistency factor establishes dynamic feedback between association and state estimation.
- An efficient alternating optimization strategy and a sliding time window are introduced to control computational complexity and maintain real-time performance.
- Extensive experiments on the KITTI and nuScenes demonstrate the superiority of the proposed method over other existing trackers, including RFS-based and FGO-based trackers, showing improved accuracy, identity consistency, occlusion recovery, and trajectory stability.

The rest of the paper is organized as follows: Section 2 explains the relevant theories and methods of 3D MOT; Section 3 introduces the proposed algorithm framework in detail; Section 4 presents and analyzes the experimental details and results; and Section 5 gives the conclusion.

## 2. Related Works

This section reviews the relevant theories and methodologies in the field of multi-object tracking (MOT), especially 3D MOT.

### 2.1. Data Association-Based MOT

In 3D MOT, data association–based methods dominate the mainstream, where tracking is decoupled into detection and association. Tracking-by-detection (TBD) is the most widely adopted paradigm in data association-based MOT.

In TBD, the trajectory state is first predicted using motion models such as the Kalman filter [16]. The predicted trajectories are then compared with current 3D detections through similarity metrics, commonly 3D Intersection over Union (3D IoU) or Mahalanobis distance. These pairwise similarities form a similarity matrix, from which optimal assignments are obtained using matching algorithms such as the Hungarian method [17] or greedy strategies. Finally, lifecycle management, including state update, trajectory initialization and termination is performed based on the association results.

Additionally, the TBD paradigm has led to a series of efficient 3D trackers. For example, AB3DMOT [2], a strong baseline, extends SORT [1] into 3D space, using the Kalman filter and 3D IoU–based association. CenterPoint [18] also adopts this simple and effective strategy, achieving strong performance through velocity prediction and greedy matching. SimpleTrack [4] further highlights the effectiveness of lightweight association mechanisms. To improve robustness, multimodal approaches incorporate image information to assist tracking, where several works [19,20,21,22] fuse 2D appearance and 3D geometric similarity to enhance data association.

Despite their efficiency, the TBD paradigm still suffers from inherent limitations. Rigid association strategies can easily cause ID switches (IDSW) or trajectory fragmentation in cases of occlusion or missed detection. Moreover, heuristic rule-based object management lacks a unified probabilistic model for handling uncertainty. These limitations indicate that, despite recent advances in practical performance, TBD and JDT methods remain largely constrained by frame-by-frame greedy decision-making and fail to fully exploit temporal information or model uncertainty in a principled manner.

Recently, joint detection and tracking (JDT) methods [23,24,25] have emerged, which jointly model detection and identity prediction, thereby better handling occlusions and missed detections. However, most JDT approaches still rely on heuristic matching or ad-hoc association rules, and may fail to fully account for the uncertainty in object existence and measurement.

Therefore, addressing these limitations requires tracking strategies that move beyond greedy, frame-by-frame decision-making and are capable of explicitly handling occlusions, missed detections, and uncertainty. By jointly leveraging information across multiple frames, such approaches can effectively reduce identity switches, mitigate trajectory fragmentation, and produce more consistent multi-object trajectories.

### 2.2. RFS-Based MOT

To overcome the limitations of the traditional TBD paradigm, random finite set (RFS) theory provides a unified probabilistic tracking framework for MOT. By representing the multi-object state as a random finite set, RFS jointly models the uncertainty in both the number and states of objects. This formulation enables Bayesian filtering to naturally address object birth and death, uncertainty in data association, missed detections, and clutter. Among various RFS filters, Bernoulli-based formulations have attracted considerable attention due to their ability to explicitly estimate both the existence probability and the state of each potential object.

Although RFS has been extensively utilized in point-target and extended-target tracking [26,27,28], its application to bounding box-based MOT has only attracted increasing attention in recent years. Pang et al. [29] demonstrated the effectiveness of multi-Bernoulli mixture (PMBM) filtering on LiDAR-based autonomous driving datasets. To improve computational efficiency, Kropfreiter et al. [30] and Shim et al. [31] investigated hypothesis management and label partitioning strategies, while Ishtiaq et al. [32] incorporated interaction-aware modeling into labeled multi-Bernoulli filters to enhance robustness in crowded environments. These studies highlight the strong capability of RFS-based approaches in uncertainty modeling, efficiency, and interaction handling. Building upon these advances, several works have further explored the application of RFS to 3D bounding box–based MOT. Representative efforts include RFS-M3 [11], which is the first to effectively apply the PMBM filter to the TBD paradigm, introducing detection confidence scores into the prediction and update processes. PTMOT [10] builds on this success by integrating and smoothing the confidence scores of tiny tracks, further improving the performance of the PMBM framework when dealing with discontinuous trajectories. GNN-PMB [12] employs a global nearest neighbor (GNN) algorithm to solve the assignment problem within the Poisson Multi-Bernoulli Filter (PMB).

However, despite the improvements in efficiency and interaction modeling seen in these recent works, most existing RFS-based MOT methods remain inherently online and rely on frame-by-frame Bayesian filtering. Such greedy decision-making limits their ability to jointly exploit temporal information over multiple frames and to revise earlier association decisions using future observations. As a result, tracking errors caused by occlusions or missed detections may accumulate and propagate over time. This limitation motivates the development of an optimization-based RFS framework that can jointly estimate object states, associations, and existence probabilities over a temporal window. Unlike conventional recursive filters, such a framework would be capable of leveraging both past and future observations to correct earlier errors, thereby improving trajectory continuity in challenging scenarios.

### 2.3. Graph Optimization-Based MOT

To address the local optimal problem in online tracking frameworks, graph optimization-based methods provide global joint optimization solutions. Graph optimization methods treat MOT as a graph-structured problem, attempting to find a temporally consistent optimal solution by globally optimizing all relevant information in a sequence. Recent studies have explored diverse applications of graph optimization in MOT, including multi-sensor fusion, multi-camera tracking, satellite video tracking, and learnable graph matching, demonstrating its versatility and ongoing development [33,34,35].

Among various graph optimization techniques, factor graph optimization (FGO) stands out for its high modeling flexibility. It encodes object states as variable nodes and expresses constraints such as observation error, motion consistency, and correlation relationships as factors that connect these nodes. While FGO has been proven effective in robotics and SLAM, its application to object tracking, particularly in 3D scenes, is still in the early stages. Wang et al. [36] significantly improved the accuracy and stability of tracking densely moving objects by applying FGO to multi-hypothesis tracking. Pöschmann et al. [14] represent 3D object detection results in point cloud scenes as a Gaussian mixture model (GMM) and perform joint optimization within a factor graph framework, implicitly addressing the data association problem. Feng et al. [15] propose a real-time 3D-LiDAR MOT method that combines a 6-degree-of-freedom acceleration and angular velocity (6-DoF CAAV) motion model, hybrid feature measurements, and sliding window-based FGO.

In summary, while graph optimization enables global trajectory consistency and corrects continuous-state estimation errors, most existing approaches still do not explicitly model uncertainties in object existence, measurement origin, or object birth and disappearance. Consequently, identity switches and trajectory fragmentation remain challenges in complex 3D MOT scenarios. This motivates the exploration of tracking strategies that can jointly leverage temporal information and reason about uncertainty, leading to more consistent and robust multi-object trajectories.

### 2.4. Summary and Motivation

Overall, recent studies in RFS-based and graph optimization-based 3D MOT have substantially improved tracking performance. Nevertheless, existing methods typically focus on either probabilistic uncertainty modeling, as employed in RFS-based filters, or global trajectory optimization, as used in graph-based approaches, and rarely integrate both within a unified framework. As a result, challenges such as identity switches, trajectory fragmentation, and incomplete uncertainty handling persist in complex scenarios.

Motivated by these observations, we propose a unified FGO-PMB framework that tightly integrates RFS-based uncertainty modeling with factor graph optimization. By representing object states, existence probabilities, and association uncertainties as optimizable variables within a factor graph, the proposed method enables joint global inference over a sliding time window, achieving robust and temporally consistent 3D multi-object tracking even under occlusion, missed detections, and cluttered environments.

## 3. Method

**Figure 1:**
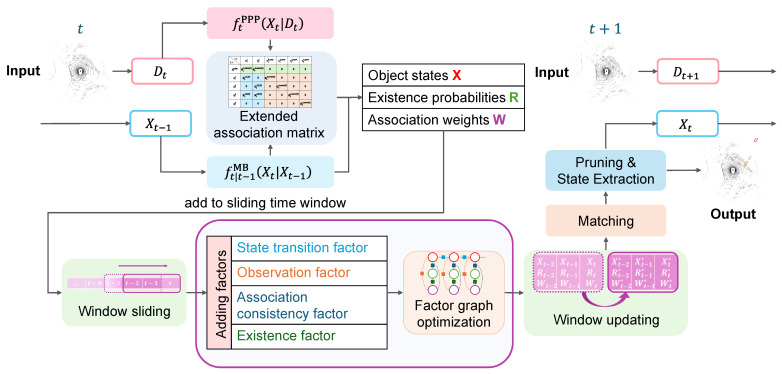
Overview of the proposed FGO-PMB framework for 3D multi-object tracking.

This section presents the proposed 3D MOT framework named FGO-PMB, which integrates RFS and FGO. The overall workflow is illustrated in Figure 1. The process operates continuously over time steps. At each current frame *t*, the system takes two inputs: the set of detections $D_{t}$ from the detector and the estimated object states $X_{t-1}$ from the previous frame. First, a Poisson Multi-Bernoulli (PMB) model is employed to predict the object states probabilistically. This involves computing the Poisson point process (PPP) intensity $f_{t}^{\mathrm{PPP}}\left(X_{t}|D_{t}\right)$ for potential birth objects and the Multi-Bernoulli (MB) spatial density $f_{t|t-1}^{\mathrm{MB}}\left(X_{t}|X_{t-1}\right)$ for surviving objects. Based on these distributions, an extended association matrix is constructed to generate three key variables: the refined object states *X*, the existence probabilities *R*, and the association weights *W*. Subsequently, these variables are collected within a sliding time window. A global optimization is performed using a factor graph to jointly solve for the optimal variables by considering multiple constraints, including state transition, observation, association consistency, and existence factors. Finally, the optimal states for the current frame are extracted through a matching and pruning process to produce the final tracking output.

### 3.1. Modeling

The proposed method uses the TBD paradigm to track multiple dynamic objects in a 3D scene online. Assume that at time *t*, there is a set of targets $T_{t}=\left\{\tau_{t}^{1},\tau_{t}^{2},\ldots ,\tau_{t}^{M_{t}}\right\}$, where $M_{t}$ denotes the number of targets at time *t*. The state vector of each target $\tau_{t}^{j}$ is defined as $x_{t}^{j}=\left\{p_{x},p_{y},p_{z},h,w,l,v_{xy},\theta ,\omega ,cls,id\right\}$. Here, $\left(p_{x},p_{y},p_{z}\right)$ represents the 3D center position of the object, (h,w,l) denotes the height, width, and length of the 3D bounding box, $v_{xy}$ is the velocity magnitude in the x−y plane, θ denotes the heading angle, ω is the heading angular velocity or turning rate, cls represents the object class, and id is the unique object identifier. Meanwhile, the detector provides a set of observations $Z_{t}=\left\{z_{t}^{1},z_{t}^{2},\ldots ,z_{t}^{N_{t}}\right\}$, where each observation $z_{t}^{i}=\left\{p_{x},p_{y},p_{z},h,w,l,\theta ,cls,s\right\}$ represents the 3D center position, size, orientation, class, and detection confidence score of each detection box, and $N_{t}$ denotes the number of detections at time *t*. Notably, any 3D detector that produces standard 3D detections can be utilized with our proposed tracker.

### 3.2. Variable Initialization

Before performing FGO, it is necessary to provide reasonable initial estimates for all variables to be optimized, including object states, existence probabilities, and association weights. Good initialization is essential for efficiently solving nonlinear optimization problems. It improves convergence speed and stability, while also helping the optimizer avoid local optima in complex scenarios. This subsection presents our initialization strategy, which is derived from the principles of RFS theory. By modeling the survival of existing objects and the emergence of new ones within a unified probabilistic framework, it enables consistent and principled initialization for subsequent graph optimization.

Specifically, for each object *j* that may survive from time t−1 to time *t*, the prediction is derived from the multi-Bernoulli representation. Assuming that it survives independently with a constant survival probability $P_{s}\in \left[0,1\right]$, its existence probability $r_{t}^{j(0)}$ at time *t* is predicted as follows:

(1) $$r_{t}^{j(0)}=P_{s}\cdot {\hat{r}}_{t-1}^{j}.$$

The corresponding state vector $x_{t}^{j}\in R^{d}$ is predicted according to a state transition function that characterizes the temporal evolution of the object based on its kinematic properties. The general state transition equation is formulated as:

(2) $$x_{t}^{j(0)}=f\left({\hat{x}}_{t-1}^{j},\Delta t\right).$$

In this work, to accurately capture the maneuvering characteristics of traffic participants, which often involve coordinated turns, we implement the function f(·) using the nonlinear Constant Turn Rate and Velocity (CTRV) model [37]. Unlike simple linear models such as Constant Velocity (CV), the CTRV model explicitly incorporates the heading angular velocity, enabling a more accurate representation of the curvilinear motion of maneuvering targets. The specific formulations of the CTRV model, presented in Equations (3)–(7), are derived by integrating the kinematic differential equations of the object state over the time interval Δt, under the assumption that both the velocity magnitude $v_{xy}$ and the heading angular velocity ω remain constant during this period. Specifically, the state transition function f(·) is formulated as:

(3) $$p_{x}^{t}=p_{x}^{t-1}+\frac{v_{xy}^{t-1}}{\omega^{t-1}}\left(\sin\left(\theta^{t-1}+\omega^{t-1}\Delta t\right)-\sin\left(\theta^{t-1}\right)\right),$$

(4) $$p_{y}^{t}=p_{y}^{t-1}+\frac{v_{xy}^{t-1}}{\omega^{t-1}}\left(-\cos\left(\theta^{t-1}+\omega^{t-1}\Delta t\right)+\cos\left(\theta^{t-1}\right)\right),$$

(5) $$v_{xy}^{t}=v_{xy}^{t-1},$$

(6) $$\theta^{t}=\theta^{t-1}+\omega^{t-1}\Delta t,$$

(7) $$\omega^{t}=\omega^{t-1},$$

where $\left(p_{x},p_{y}\right)$ donates the 2D center position in the x−y plane, $v_{xy}$ is the velocity magnitude, θ is the heading angle, ω is the heading angular velocity, and Δt represents the time interval.

To accommodate the potential new targets within a unified framework, the method models them using an observation-driven Poisson Point Process (PPP). Specifically, at time *t*, a potential new target hypothesis is generated for each of the $N_{t}$ observations ${\left\{z_{t}^{i}\right\}}_{i=1}^{N_{t}}$ in the current frame. These potential new target hypotheses are indexed consecutively after the $N_{t}$ existing objects, forming a unified and expanded set of object hypotheses. The initial state $x_{t}^{j}$ of each potential new target hypothesis is directly initialized from its corresponding observation $z_{t}^{i}$:

(8) $$x_{t}^{M_{t}+i\left(0\right)}=\mathrm{Init}\left(z_{t}^{i}\right),$$

where $M_{t}$ denotes the number of surviving objects predicted at the current time step *t*, and the initialization function Init(·) assigns the detected position and the heading angle directly to the position and angle component of the state, while the velocity and the heading angular velocity are initially set to zero. The existence probability $r_{t}^{M_{t}+i\left(0\right)}$ is initialized to an intermediate value reflecting maximum uncertainty (e.g., 0.5).

After initializing the survival objects and potential new targets, prior information on data association is incorporated into the FGO. Unlike traditional methods that treat association as an independent decision step, our method uses PMB to estimate the association probabilities of each observation $z_{t}^{i}$ with all potential target sources and incorporates the probabilities into the optimizer as a soft prior. Specifically, for the i-*th* observation and the j-*th* object hypothesis, the initial association weight $w_{t}^{i,j(0)}$ is given by:

(9) $$w_{t}^{i,j(0)}=\left\{\begin{array}{cc} r_{t}^{j(0)}\cdot p_{D}\cdot N\left(z_{t}^{i}|Hx_{t}^{j(0)},R\right), & \mathrm{if}\;j\in [0,M_{t}-1];\\ s_{i}\cdot K\left(z_{t}^{i}\right), & \mathrm{if}\;j=M_{t}-1+i;\\ 0, & \mathrm{otherwise},\; \end{array}\right.$$

where N represents the Gaussian likelihood function, *R* is the observation noise covariance, $s_{i}$ is the detection confidence score, and $K(z_{t}^{i})$ is the Poisson intensity representing the likelihood of new object’s occurrence. The proposed equation defines three cases: (1) For surviving objects, the weight is given by the product of their existence probability $r_{t}^{j}$, detection probability $p_{D}$, and the Gaussian observation likelihood function $N(z_{t}^{i}|Hx_{t}^{j},R)$, where *H* is the observation matrix and *R* is the observation noise covariance; (2) For potential new objects, the weight is defined by the product of the observation confidence $s_{i}$ and the Poisson intensity $K(z_{t}^{i})$; (3) For all other cases, the association weight is set to zero. This covers associations rejected by the gating mechanism as well as invalid pairings involving potential new objects. Specifically, each potential new object is uniquely generated from a single observation; thus, the *i*-th observation corresponds exclusively to the new object hypothesis indexed by $j=M_{t}-1+i$. Any association with $j\neq M_{t}-1+i$ is invalid, as it implies associating an observation with a new object hypothesis generated by another measurement.

Then, the association weights of each observation are normalized as:

(10) $${\tilde{w}}_{t}^{i,j(0)}=\frac{w_{t}^{i,j(0)}}{\sum_{k=1}^{M_{t}+N_{t}}w_{t}^{i,k(0)}},$$

which ensures that for any observation *i*, the total probability that it originates from all possible sources sums to 1, i.e., $\sum_{j}{\tilde{w}}_{t}^{i,j(0)}=1$. The normalized association weights are subsequently used as soft priors in the FGO.

**Figure 2:**
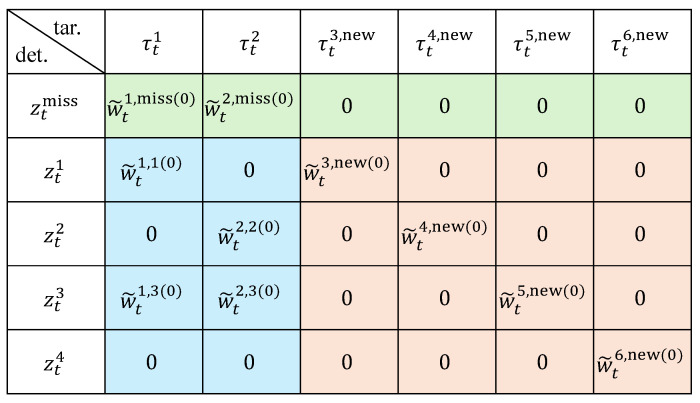
Structure of the extended association matrix used in FGO-PMB.

Figure 2 illustrates the final structure of the extended association matrix, whose elements are populated according to Equation (10). Specifically, the rows of this matrix not only represent all actual observations $z_{t}^{j}$ at the current time step, but also include an additional row $z_{t}^{miss}$ (the first row, shown in green) corresponding to the missed-detection hypothesis, which accounts for undetected trajectories. The columns correspond to existing targets (blue) and new target candidates (red), with weights between unrelated pairs set to 0. This matrix thus provides a unified representation of the initial likelihood of association between observations and all potential targets.

### 3.3. Factor Graph

To jointly model the probabilistic dependencies among multiple targets and the temporal consistency of individual trajectories, a factor graph is employed as a unified optimization framework. In the FGO framework, variable nodes represent the variables to be optimized, while factor nodes encode probabilistic constraints. By applying this framework to MOT, the tracking problem is formulated as a global objective that aggregates the costs of all factors, allowing joint optimization over multiple targets and time steps. Section 3.3.1 details the construction of individual factors, while Section 3.3.2 presents the optimization strategy for solving the graph.

#### 3.3.1. Factor Graph Construction

**Figure 3:**
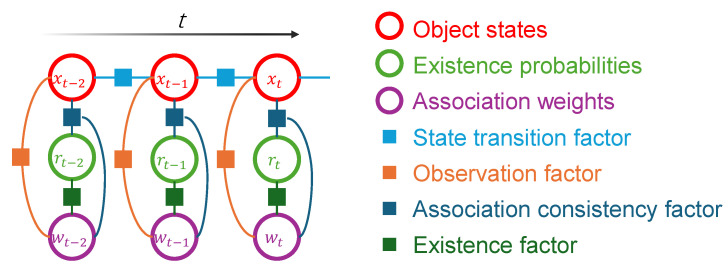
Factor graph formulation for joint optimization of states, existence, and associations.

Based on the requirements of object motion and observation modeling, four factors are formulated, including the state transition factor, observation factor, association consistency factor, and existence factor. These factors jointly characterize the global probabilistic relationships among the three variables, including object state *x*, data association *w*, and existence probability *r*. The connections between these factors and variables are illustrated in Figure 3, which provides a unified formulation linking these variables across time steps to enable global joint optimization over all objects.

(a) State transition factor

The state transition factor ensures temporal consistency in an object’s motion by constraining the relationship between the state variables of consecutive frames. Both the previous state $x_{t-1}^{j}$ and the current state $x_{t}^{j}$ are treated as optimization variables. The motion model generates a predicted state based on $x_{t-1}^{j}$, and the Mahalanobis distance is used to quantify the residual between this predicted state and the variable $x_{t}^{j}$. The error function of the state transition factor is defined as follows:

(11) $$\varepsilon_{\mathrm{trans}}^{t,j}=∥x_{t}^{j}-f\left(x_{t-1}^{j}\right){∥}_{Q}^{2},$$

where f(·) denotes the state transition function, which follows the CTRV nonlinear motion model given in Equations (3)–(7). *Q* denotes the process noise covariance matrix, which represents the uncertainty of the state transition process.

(b) Observation factor

The observation factor imposes consistency constraints between the object state variables and sensor observations, thereby guiding the estimated state to stay closer to the most relevant observations. This factor maps the object state $x_{t}^{j}$ to the observation space through the observation model, and measures the residual between the object state and the actual observation using the Mahalanobis distance. The association weight variables $w_{t}^{i,j}$ act as a soft gating mechanism, amplifying the effect of high-confidence associations on the optimization while suppressing low-confidence ones, thereby ensuring that each state is guided by the most relevant observations. The error function of the observation factor is defined as follows:

(12) $$\varepsilon_{\mathrm{obs}}^{t,i,j}=w_{t}^{i,j}\cdot {∥z_{t}^{i}-Hx_{t}^{j}∥}_{R}^{2},$$

where *H* is the observation matrix and *R* is the observation error covariance. The variable $w_{t}^{i,j}\in \left[0,1\right]$ serves as a soft association weight between the state $x_{t}^{j}$ and the observation $z_{t}^{i}$. A higher $w_{t}^{i,j}$ enforces a stronger constraint linking the state to the observation, whereas a value close to zero means the observation exerts negligible influence on the state estimate.

(c) Association consistency factor

The association consistency factor imposes a probabilistic constraint that links the association weight to the current state estimate. It aims to ensure that the association decisions remain consistent with the observation likelihood. The likelihood is derived from the estimated state during optimization. Specifically, the residual of the association consistency factor is defined as follows:

(13) $$\varepsilon_{\mathrm{assoc}}^{t,i,j}={\left(w_{t}^{i,j}-{\tilde{w}}_{t}^{i,j}\right)}^{2},$$

where ${\tilde{w}}_{t}^{i,j}$ is computed from the observation likelihood $N(z_{t}^{i}|Hx_{t}^{j},R)$ between the current state $x_{t}^{j}$ and the observation $z_{t}^{i}$, together with the existence probability $r_{t}^{j}$, as given in Equation (10). Importantly, unlike the initialization ${\tilde{w}}_{t}^{i,j(0)}$, ${\tilde{w}}_{t}^{i,j}$ is updated with the current state and existence variables and therefore changes dynamically during optimization. This factor encourages the variable $w_{t}^{i,j}$ to be consistent with the ${\tilde{w}}_{t}^{i,j}$ inferred from the current state. This consistency establishes a two-way coupling in which the observation likelihood influences the state estimate, and the updated state further refines the association weights.

(d) Existence factor

The existence factor models both the temporal evolution of an object’s existence probability and the consistency of its association with observations. First, the object’s existence probability is modeled by a prior dynamic process of survival and extinction. Assuming that the probability of an object existing at time *t* is $r_{t}^{j}$ and at the previous time is $r_{t-1}^{j}$, its temporal evolution can be expressed as:

(14) $$\varepsilon_{\mathrm{evol}}^{t,j}={\left(r_{t}^{j}-P_{s}\cdot r_{t-1}^{j}\right)}^{2},$$

where $P_{s}$ denotes the survival probability, the existence probability $r_{t}^{j}$ is encouraged to evolve smoothly over time. Additionally, $r_{t}^{j}$ remains consistent with the association result of the current observation. Accordingly, the association support consistency constraint can be expressed as:

(15) $$\varepsilon_{\sup}^{t,j}={\left(r_{t}^{j}-\sum_{i}w_{t}^{i,j}\right)}^{2}.$$

Finally, the overall form of the existence factor is given by

(16) $$\varepsilon_{\mathrm{exist}}^{t,j}=\lambda_{1}\cdot \varepsilon_{\mathrm{evol}}^{t,j}+\lambda_{2}\cdot \varepsilon_{\sup}^{t,j},$$

where $\lambda_{1}$ and $\lambda_{2}$ are balance coefficients that control the relative contributions of $\varepsilon_{\mathrm{evol}}^{t,j}$ and $\varepsilon_{\sup}^{t,j}$, respectively. This factor improves the robustness of continuous object tracking under occlusion or poor observations by jointly constraining temporal priors and associations with observations.

#### 3.3.2. Optimization Strategy

After constructing the complete factor graph, the global objective function for joint optimization is formulated as the sum of all factor errors, which is defined as follows:

(17) $$\varepsilon_{\mathrm{total}}=\sum_{j}\left[\varepsilon_{\mathrm{trans}}^{t,j}+\varepsilon_{\mathrm{exist}}^{t,j}+\sum_{i}\left(\varepsilon_{\mathrm{obs}}^{t,i,j}+\varepsilon_{\mathrm{assoc}}^{t,i,j}\right)\right].$$

However, direct joint optimization of this objective function, which involves state variables, existence probabilities, and association variables, results in a highly coupled and challenging non-convex problem. Specifically, a key challenge is the mutual dependency: association variables are computed based on the states and existence probabilities of all objects, while the optimization of states and existence probabilities in turn depends on the association variables. This circular dependence leads to a highly dense Jacobian matrix during optimization, thereby markedly increasing the computational complexity of the optimizer.

**Figure 4:**
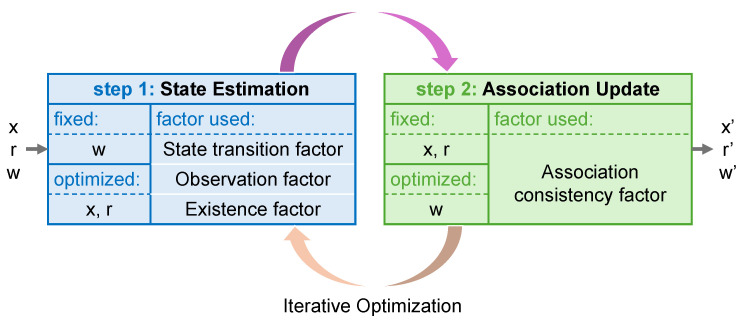
Alternating optimization strategy of FGO-PMB.

To this end, an alternating optimization strategy is proposed in our work to decompose the overall optimization problem into two simpler subproblems. Specifically, one is to fix the association weights while optimizing the object state and existence probability, and another is to analytically compute the association weights based on the currently optimized state and existence probability. These two steps are alternated in an iterative process, with refinement ensured by a soft update strategy. The process is shown in Figure 4, which facilitates the collaborative convergence of state estimation and data association.

Specifically, at each iteration, first, the association variables are fixed to decouple the objective function into subproblems involving only state and existence probabilities. A factor graph containing only state transition factors, observation factors, and existence factors is constructed, and the current optimal estimates of state and existence probabilities are obtained using a nonlinear optimizer, such as Levenberg-Marquardt. Then, the latest optimized estimates of the state and existence probabilities are fixed, and the association weights are recomputed analytically outside of the factor graph as defined in Equations (9) and (10) in Section 3.2. Although the association consistency factors are no longer explicitly modeled within the factor graph, their constraint effects are effectively preserved and realized through the update process outside the graph. This alternating optimization process is iterated until the objective function converges or the maximum number of iterations is reached.

### 3.4. Post Processing

Rather than optimizing over an ever-growing trajectory history, the proposed framework adopts a sliding time window mechanism with a fixed length *L*, which controls computational complexity while preserving tracking quality. All joint optimizations are restricted to the sliding time window. Consequently, at any time *t*, the global objective function is expressed as the sum of the costs over all frames within the window, defined as follows:

(18) $$\varepsilon_{\mathrm{sliding}\_\mathrm{window}}=\sum_{k=t-L+1}^{t}\varepsilon_{\mathrm{total}}^{k}.$$

After reaching convergence or the maximum number of iterations within the current window [t−L+1,t], the system proceeds with subsequent post-processing steps, including output, trajectory management, and window update.

#### 3.4.1. Data Association

After FGO yields continuous association weight matrices over the sliding window, the matrix at the current time *t* is extracted and converted to discrete associations using the Hungarian or greedy algorithm.

#### 3.4.2. Object State Output

Once the optimal matching solution at time *t* is determined, the final tracking output is obtained by retaining only the trajectories successfully associated with observations. Specifically, targets with an existence probability greater than the output threshold $thr_{out}$ are considered high-confidence, and their estimated states are reported as the outputs of the current frame.

#### 3.4.3. Object Lifecycle Management

The object lifecycle management mechanism is entirely based on the existence probability in RFS theory, rather than heuristic counters, enabling more robust handling of object birth and death.

The specific management rules are as follows: for each object $\tau_{t-L+1:t}^{j}$ within the sliding time window, including potential newly born targets, survivability is assessed based on its existence probability $r_{t-L+1:t}^{j}$. For a newly generated object hypothesis from an observation, if $r_{t-L+1:t}^{j}$ exceeds the output threshold $thr_{\mathrm{out}}$, it is recognized as a new trajectory. For any object *j*, if $r_{t-L+1:t}^{j}$ falls below the deletion threshold $thr_{\mathrm{del}}$, the object is regarded as lost; if the loss persists for more than $T_{l}$ moments, the object is permanently removed and will no longer participate in subsequent optimization.

#### 3.4.4. Window Update

After completing the optimization and lifecycle management for the current window [t−L+1,t], the window advances by one time step. Specifically, all variables and factors at the earliest time t−L+1 within the window are removed, the surviving targets at time *t* are predicted to obtain the prior at t+1, the new observations $Z_{t+1}$ are introduced, and potential new targets are initialized. At this point, the factor graph covering the updated window [t−L+2,t+1] is constructed and ready for the next round of optimization.

Algorithm 1 summarizes the proposed 3D MOT framework and provides the pseudo-code of its online tracking procedure.
**Algorithm 1:** 3D Multi-Object Tracking based on PMB and FGO
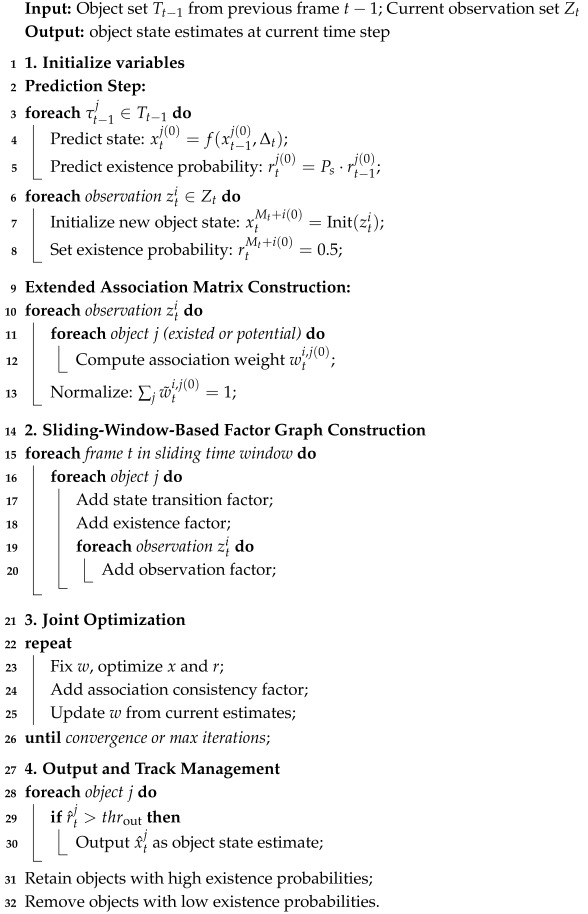


## 4. Experiments

This section presents a comprehensive experimental evaluation of the proposed 3D MOT framework. The experiments are designed to validate the effectiveness, robustness, and real-time performance of our method.

### 4.1. Settings

This section describes the experimental settings used to evaluate the proposed FGO-PMB framework. Specifically, we introduce the datasets, evaluation metrics, baseline methods, and implementation details to ensure fair and comprehensive comparisons with existing state-of-the-art tracking approaches.

#### 4.1.1. Datasets

**KITTI**: Collected in urban environments in Germany using a 64-line LiDAR and cameras, this dataset provides 10 Hz synchronized annotations. It contains 21 training and 29 testing sequences, focusing on three categories: cars, pedestrians, and cyclists. With dense and continuous annotations, it is well-suited for validating short-term tracking algorithms and multi-modal fusion.

**nuScenes**: Collected in Boston and Singapore, the dataset covers complex urban scenes and diverse weather conditions. It is captured using a 32-line LiDAR and multi-modal sensors, with data recorded at 20 Hz while annotations are provided at 2 Hz. The dataset consists of 1000 20-s driving scenes and is labeled with 23 object categories (including static objects), supporting long-term tracking and multi-modal fusion.

#### 4.1.2. Evaluation Metrics

To ensure a fair and standardized comparison, all experiments on KITTI follow the official evaluation protocol of the KITTI tracking benchmark, which is based on the HOTA [38] and CLEAR [39] MOT metrics. Specifically, we report higher order tracking accuracy (HOTA), multiple object tracking accuracy (MOTA), multiple object tracking precision (MOTP), the numbers of true positives (TP), false positives (FP), identity switches (IDSW), trajectory fragments (FRAG), as well as the mostly tracked ratios (MTR) and mostly lost ratios (MLR). In addition, we measure the runtime efficiency using frames per second (FPS).

For the nuScenes tracking evaluation, we follow the official metrics and report Average MOTA (AMOTA), Average MOTP (AMOTP), MOTA, Recall, IDSW, and FRAG. Note that the definition of MOTP in nuScenes differs from KITTI. NuScenes AMOTP/MOTP measure the average center-distance error (lower is better), while a higher MOTP indicates better performance in KITTI.

#### 4.1.3. Baseline Methods

To comprehensively evaluate the proposed tracking method FGO-PMB, we compared it with representative baseline methods. These extreme methods cover the current mainstream tracking frameworks, including mainstream tracking paradigm methods, RFS-based methods, graph theory-based methods, and multi-modal methods. The selected baseline methods include: AB3DMOT [2], Probabilistic3DMM [3], FG-3DMOT [14], EagerMOT [21], GNN-PMB [12], PolarMOT [40], CasTrack [41,42], VirConvTrack [42,43], 3DMLA [44], EAFFMOT [45], UG3DMOT [8], MMF-JDT [46], and Co-MOT [47]. Section 4.2 and Section 4.3 present the quantitative comparison results and qualitative comparison results respectively.

#### 4.1.4. Implementation Details

All experiments are conducted on a computing platform with an Intel Core i9-12900KF CPU, and the proposed framework is implemented in Python 3.8.18. For fair comparison, the hyperparameters of baseline methods fully follow their original papers or official implementations.

### 4.2. Quantitative Analysis

To quantitatively evaluate the effectiveness of the proposed method, comparative experiments are conducted on several representative 3D multi-object tracking methods on the KITTI and nuScenes tracking datasets.

Table 1 compares the 3D MOT performance of different methods on the KITTI validation set using the Casa [41] and VirConv [43] detectors. Underlined values indicate the best results with Casa, and **bold** values indicate the best with VirConv. Using the Casa detector, UG3DMOT achieves slightly higher HOTA and MOTA. However, our method achieves the highest TP(7917) and best trajectory-level metrics (MTR = 89.19%, MLR = 0.54%), indicating better target recovery and trajectory completeness. Using the VirConv detector, our method obtains the best HOTA (85.58%) and lowest IDSW(3), while maintaining near-optimal MOTA and MOTP. It also achieves a low FRAG value and the best MLR (0.54%), indicating robust tracking stability. Overall, these results demonstrate that the proposed unified FGO framework achieves competitive performance in terms of both accuracy and trajectory consistency under both detectors.

Table 2 reports the quantitative results on the KITTI test set. The **bold**, underlined, and *italic* values denote the first, second, and third best results for each metric, respectively. Compared with ten representative 3D MOT and multi-modal MOT methods from the past five years, our method shows strong overall competitiveness. Specifically, our method achieves 79.35% on HOTA, 88.24% on MOTA, and 86.54% on MOTP, ranking third, second, and third, respectively.

Notably, compared with FG-3DMOT, the representative FGO-based tracker, our method yields substantially higher accuracy across major evaluation metrics. This performance gain primarily stems from the fact that FG-3DMOT relies on hard data association, whereas our method adopts probabilistic association and joint optimization within a unified factor graph. As a result, the proposed method is more robust to detection noise and occlusions.

Furthermore, our method achieves the highest TP and lowest FP, which directly contributes to improved tracking accuracy. This improvement over other trackers can be attributed to the explicit modeling of object motion and uncertainty, enabling more reliable recovery of true object instances under challenging conditions.

Additionally, it achieves the best MTR and the lowest MLR, indicating higher trajectory completeness and tracking persistence. In contrast, greedy or frame-by-frame association methods such as AB3DMOT and PolarMOT are more prone to premature trajectory termination in complex scenes. Meanwhile, IDSW and FRAG remain at low levels, further demonstrating the effectiveness of the proposed framework in preserving trajectory integrity.

As shown in Table 3, our method achieves superior performance across all metrics on the nuScenes validation set. Noteworthy, compared to the GNN-PMB method, which is also based on RFS modeling, our algorithm improves performance on AMOTA by approximately 0.4% and significantly reduces AMOTP by 5.3%, demonstrating a superior advantage in localization accuracy. This improvement is primarily attributed to the proposed joint FGO strategy. Whereas GNN-PMB performs sequential, frame-by-frame association inference, our method enforces motion and observation consistency across multiple frames through global optimization, thereby effectively reducing localization error.

Furthermore, the lowest IDSW and FRAG values indicate stronger trajectory continuity. This suggests that explicitly modeling uncertainty and persistence helps maintain stable associations in noisy and crowded scenarios.

Table 4 presents a detailed runtime analysis of the proposed FGO-PMB on the KITTI and nuScenes datasets. In addition to the per-frame average processing time, we report statistical metrics including the minimum, maximum, and standard deviation to assess runtime stability. Notably, the real-time compliance rate reported in Table 4 shows that 95.06% of frames on KITTI and 91.59% on nuScenes are processed within their respective real-time limits (100 ms and 50 ms, corresponding to the 10 Hz and 20 Hz LiDAR input rates), demonstrating the practical feasibility of the proposed method.

**Figure 5:**
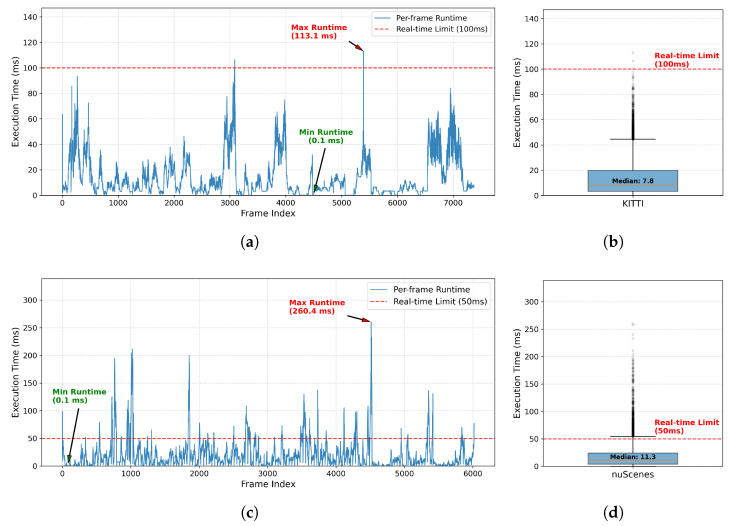
Visualization of runtime stability on KITTI and nuScenes datasets, illustrating frame-wise execution time fluctuations and statistical distribution relative to real-time constraints. (**a**) KITTI—Runtime fluctuations over continuous frames; (**b**) KITTI—Runtime distribution statistics; (**c**) nuScenes—Runtime fluctuations over continuous frames; (**d**) nuScenes—Runtime distribution statistics.

To further analyze runtime behavior, Figure 5 illustrates the frame-wise execution time fluctuations and the statistical distribution. As shown in the time-series plots (Figure 5a,c), occasional execution time spikes appear in highly crowded scenes, which correspond to worst-case scenarios. This phenomenon is expected because the computational complexity of factor graph optimization increases with the number of active objects. Nevertheless, such extreme cases occur infrequently. The box plots (Figure 5b,d) reveal consistently low median runtimes (7.8 ms for KITTI and 11.3 ms for nuScenes), indicating that the framework operates efficiently in the vast majority of scenarios.

Overall, these results demonstrate that, despite a few fluctuations in optimization-based approaches, the proposed framework maintains stable real-time performance while achieving a favorable balance among tracking accuracy, trajectory continuity, robustness, and online efficiency.

### 4.3. Qualitative Analysis

**Figure 6:**
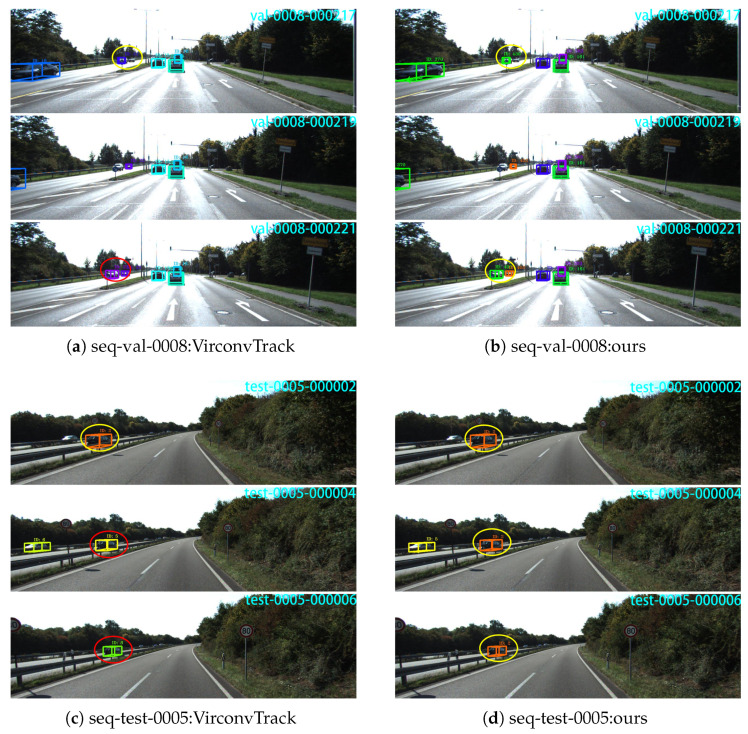
The qualitative visualization comparison between VirConvTrack (**a**,**c**) and the proposed method (**b**,**d**) on two typical sequences is shown. The experimental scenarios in (**a**,**b**) are taken from the KITTI validation set sequence 0008, and the experimental scenarios in (**c**,**d**) are taken from the KITTI test set sequence 0005. Yellow circles indicate correct tracking, and red circles indicate incorrect tracking.

As VirConvTrack achieves the best overall tracking performance among the compared baseline methods in Table 2, it is selected as a representative strong baseline for qualitative comparison. Figure 6 shows the qualitative comparison results between VirConvTrack ((a) and (c)) and our method ((b) and (d)) on two KITTI sequence segments. As shown in subfigures (a) and (b), for sequence *val-0008*, VirConvTrack fails to maintain the correct target identity after a missed detection, resulting in trajectory fragmentation and ID switches. In contrast, our method successfully recovers the association and achieves continuous trajectory tracking, benefiting from the global optimization constraints introduced by the factor graph framework. In addition, as shown in subfigures (c) and (d), for sequence *test-0005*, VirConvTrack experiences frequent ID switches when the target moves at high speed, while our method achieves stable and consistent trajectories, indicating that incorporating FGO under the RFS-based framework effectively improves tracking continuity and identity preservation, even under challenging high-speed motion scenarios.

### 4.4. Ablation Experiment

To further verify the contribution of each factor in the unified FGO framework, an ablation experiment was designed to evaluate their specific impact on the tracking performance by removing different factors separately. The experiment was conducted on the KITTI validation set for the car category. The results are presented in Table 5, where (a), (b), (c) and (d) represent the state transition factor, observation factor, existence factor and association consistency factor respectively.

The complete model (containing all four factors) achieves the highest HOTA (85.58%) and MOTA (90.85%). Removing any single key factor significantly leads to a noticeable performance drop. Specifically, removing the observation factor (b) results in a substantial decrease in HOTA and MOTA, indicating that observation constraints play a crucial role in accurate localization and data association. Removing the state transition factor (a) or the existence factor (c) leads to trajectory instability and an increase in fragmentation. Removing the association consistency factor (d) increases IDSW, demonstrating its critical role in maintaining temporal identity continuity. In summary, the results demonstrate that all four factors collaboratively contribute to improving the tracking stability and robustness of tracking.

To evaluate the impact of the iteration number in the alternating optimization on tracking performance, we conducted an ablation study by varying the maximum number of optimization iterations. The results are presented in Table 6.

The results show that when the number of iterations is set to 2, the model achieves the best balance between tracking accuracy (HOTA, MOTA) and stability (IDSW, FRAG) while maintaining a high processing speed (FPS). Fewer iterations result in the optimization failing to sufficiently propagate correlation information, leading to unstable associations and more ID switches. Conversely, more iterations lead to drops in performance and inference rate, indicating that the model may be overfitting.

To evaluate the impact of the length of the sliding time window on optimization performance, ablation experiments were conducted, as shown in Table 7. As the window length *L* increases from 2 to 4, HOTA, MOTA, and MOTP exhibit significant improvement, indicating that moderately increasing the window length helps improve correlation stability. However, when *L* is further increased to 5 or 6, the accuracy actually decreases slightly, while the processing speed FPS decreases significantly, suggesting that an excessively long window introduces unnecessary computational burden without notable benefit. Considering both accuracy and real-time performance, a window length of L=4 provides the best trade-off and is thus adopted as the default setting.

To obtain $\lambda_{1}$ and $\lambda_{2}$ in Equation (16) that maximize performance, an ablation experiment was designed, and the results are presented in Table 8. The results indicate that optimal performance is achieved when $\lambda_{1}=5$ and $\lambda_{2}=1$.

### 4.5. Failure Case Analysis

**Figure 7:**
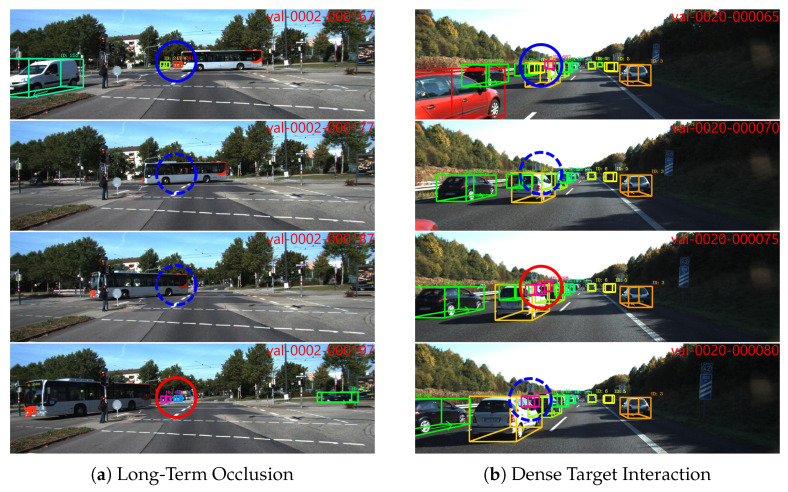
Representative failure cases of the proposed FGO-PMB framework on the KITTI validation set. The blue circles highlight the targets for which tracking failures occur. Dashed bounding boxes indicate missed detections, while red bounding boxes denote ID switches.

Despite the overall robustness of the proposed FGO-PMB framework, failure cases can still arise under challenging conditions. Figure 7 illustrates two representative failure scenarios observed on the KITTI validation set.

The first case shown in Figure 7a illustrates a long-term complete occlusion scenario, where a large bus fully occludes two targets for nearly 30 consecutive frames. Due to the absence of reliable observations over an extended period, the associated object hypotheses gradually lose support within the sliding window, leading to trajectory fragmentation and ID switches when the targets reappear. This failure case highlights the limitation of maintaining target identity under prolonged full occlusion with limited temporal context.

The second case shown in Figure 7b occurs in a highly crowded traffic scene with dense target interactions. Frequent mutual occlusions and close spatial proximity significantly increase the difficulty of data association. Although the FGO enforces global consistency, the alternating optimization strategy can converge to a suboptimal solution when multiple association hypotheses have similar posterior probabilities. Consequently, temporary tracking interruptions occur for both targets, and one target experiences an ID switch within a short time.

These failure cases reflect the inherent challenges of long-term occlusion handling and dense multi-object interaction, motivating the development of more robust optimization strategies and long-term association mechanisms.

## 5. Conclusions

In this work, we proposed FGO-PMB, a unified probabilistic framework for LiDAR-based 3D multi-object tracking that tightly integrates the PMB Filter from RFS theory with FGO. The core contribution is the first unified modeling of object states, existence probabilities, and association uncertainties from the RFS framework as optimizable variable nodes in a factor graph, enabling a unified representation of multiple sources of uncertainty. To this end, we formulate a set of probabilistic factors, including state transition, observation, existence, and association consistency for joint optimization, and propose an efficient alternating optimization strategy based on a sliding time window to achieve real-time performance. By combining the uncertainty modeling capability of RFS with the global optimization strength of FGO, and unifying state estimation, data association, and object lifecycle management within a joint optimization framework, the proposed method delivers temporally consistent and uncertainty-aware estimation across continuous LiDAR scans.

Extensive experiments on the KITTI and nuScenes datasets demonstrate that FGO-PMB achieves robust and consistent tracking performance, particularly in challenging scenarios with high-speed target motion. On the KITTI validation set, FGO-PMB achieves the highest HOTA (85.58) and the lowest number of IDSW (3) when using the VirConv detector, while maintaining competitive MOTA and MOTP values. These results confirm the effectiveness of the proposed framework in preserving target identity and maintaining trajectory continuity under challenging motion dynamics. On the nuScenes dataset, FGO-PMB further achieves the highest AMOTA (0.623) and MOTA (0.532), along with the lowest IDSW (487) and the highest recall (0.644), demonstrating strong robustness and scalability in large-scale, complex urban environments.

Despite the promising results, the proposed framework has several limitations. Specifically, the alternating optimization strategy improves computational efficiency by decomposing the original joint problem, but may not always converge to the global optimum in highly complex scenarios with dense targets or long-term occlusions. Moreover, the computational cost of FGO increases with larger sliding windows and higher target densities. Motivated by these limitations, future work will focus on two main directions for LiDAR-based 3D MOT. Firstly, we plan to develop more efficient optimization strategies to further reduce computational latency, especially in large-scale and high-density scenarios. Secondly, we aim to enhance long-term tracking consistency under challenging conditions such as extended occlusions, low-reflectivity targets, and high-speed object motion.

## Figures and Tables

**Table 1:** Comparison of the tracking performance on the val set of KITTI.

Method	Detector	HOTA ↑	MOTA ↑	MOTP ↑	TP ↑	FP ↓	MTR ↑	MLR ↓	IDSW ↓	FRAG ↓
UG3DMOT	Casa	83.73	90.46	89.92	7900	319	87.03	1.08	1	51
	VirConv	85.55	**91.06**	**91.99**	7907	**273**	89.19	1.08	4	**45**
CasTrack	Casa	82.59	88.66	90.11	7770	335	83.24	1.62	6	97
VirConvTrack	VirConv	85.43	89.93	91.82	**7936**	389	**90.27**	**0.54**	12	73
Ours	Casa	82.95	89.26	89.69	7917	434	89.19	0.54	5	75
	VirConv	**85.58**	90.85	91.61	7918	303	89.73	**0.54**	**3**	59

**Table 2:** Comparison of the tracking performance on the KITTI 2D MOT car tracking benchmark.

Method	Year	Modality	HOTA ↑	MOTA ↑	MOTP ↑	TP ↑	FP ↓	MTR ↑	MLR ↓	IDSW ↓	FRAG ↓
AB3DMOT	2020	3D	69.99	83.61	85.23	29,849	4543	66.92	9.08	113	206
FG-3DMOT *	2020	3D	-	83.74	84.64	-	-	68.00	9.85	9	375
EagerMOT	2021	2D/3D	74.39	87.82	85.69	30,895	3497	76.15	*2.46*	239	390
PolarMOT	2022	3D	75.16	85.08	85.63	31,724	2668	80.92	*2.46*	462	599
VirConvTrack	2023	3D	**81.87**	**90.24**	**86.82**	31,744	2648	83.08	11.69	**8**	**77**
3DMLA	2023	3D	75.65	85.03	84.93	31,907	3797	70.77	5.85	39	367
EAFFMOT	2024	3D	72.28	84.77	85.08	30,446	3946	70.92	8.31	107	287
UG3DMOT	2024	3D	78.60	87.98	86.56	31,399	2993	79.08	5.38	*30*	360
MMF-JDT	2025	2D/3D	79.52	*88.06*	86.24	32,075	2317	80.15	2.62	*37*	363
Co-MOT	2025	3D	75.76	85.29	85.62	*31,756*	*2636*	*81.54*	2.31	420	599
Ours	-	3D	*79.35*	88.24	*86.54*	**32,314**	**2078**	**84.77**	**2.15**	60	*243*

* The online evaluation results for this method are obtained from the paper [14], while the results for other methods are obtained from the KITTI tracking benchmark https://www.cvlibs.net/datasets/kitti/eval_tracking.php, accessed on 3 December 2025.

**Table 3:** Comparison of the tracking performance on the val set of nuScenes.

Method	Detector	Modal	AMOTA ↑	AMOTP ↓	MOTA ↑	Recall ↑	IDSW ↓	FRAG ↓
AB3DMOT	MEGVII	3D	0.509	0.934	0.453	0.558	1138	742
Probabilistic3DMM	MEGVII	3D	0.561	0.800	0.483	0.606	679	606
EAFFMOT	MEGVII	3D	0.595	0.744	0.511	0.622	565	481
GNN-PMB	MEGVII	3D	0.619	0.716	-	-	508	372
Ours	MEGVII	3D	**0.623**	**0.663**	**0.532**	**0.644**	**487**	**320**

**Bold** values indicate the best performance among all compared methods for each metric.

**Table 4:** Runtime analysis of the proposed FGO-PMB on KITTI and nuScenes.

Module	KITTI (ms/frame)	nuScenes (ms/frame)
Preprocessing & Prediction	0.9	1.0
Factor Graph Construction	1.6	2.9
Factor Graph Optimization	12.0	27.5
Data Association	0.1	0.2
State Extracting & Pruning	0.1	0.1
Total Average	14.7	31.7
Minimum	0.1	0.1
Maximum	113.1	260.4
Standard Deviation	15.99	25.8
Real-time Compliance Rate	95.06%	91.59%

**Table 5:** Contribution analysis of different factors in the FGO-PMB framework.

a	b	c	d	HOTA ↑	MOTA ↑	MOTP ↑	IDSW ↓	FRAG ↓
×	✓	✓	✓	84.38	90.15	**91.93**	52	62
✓	×	✓	✓	50.70	56.00	80.55	1189	275
✓	✓	×	✓	83.06	86.91	91.47	5	**26**
✓	✓	✓	×	85.42	90.67	91.55	7	60
✓	✓	✓	✓	**85.58**	**90.85**	91.61	**3**	59

**Bold** values indicate the best performance among different factor combinations for each metric.

**Table 6:** Ablation on the maximum number of iterations in alternating optimization.

Iterations	HOTA ↑	MOTA ↑	IDSW ↓	FRAG ↓	FPS ↑
1	83.28	90.41	11	68	**79**
2	**85.58**	**90.85**	**3**	**59**	63
3	83.74	90.49	10	63	40
4	82.76	90.19	12	71	20

**Bold** values indicate the best performance under different iteration settings for each metric.

**Table 7:** Ablation study on different sliding time window lengths.

*L*	HOTA ↑	MOTA ↑	MOTP ↑	IDSW ↓	FRAG ↓	FPS ↑
2	51.97	65.50	84.08	634	329	**95**
3	84.52	90.63	90.83	10	63	73
4	**85.58**	**90.85**	**91.61**	**3**	**59**	63
5	84.74	90.79	91.51	5	62	42
6	84.77	90.79	91.53	5	62	34

**Bold** values indicate the best performance under different sliding window lengths for each metric.

**Table 8:** Impact of the weights $\lambda_{1}$ and $\lambda_{2}$ in Equation (16) on tracking performance.

$\lambda_{1}$	$\lambda_{2}$	HOTA ↑	MOTA ↑	MOTP ↑	IDSW ↓	FRAG ↓
1	1	84.74	90.57	91.63	4	62
1	3	84.53	90.36	**91.64**	7	63
1	5	84.19	90.32	**91.64**	8	63
1	7	84.29	90.42	**91.64**	8	63
3	1	84.81	90.76	91.62	4	62
5	1	**85.58**	**90.85**	91.61	**3**	59
7	1	84.78	89.78	91.56	4	**49**

**Bold** values indicate the best performance under different weight settings for each metric.

## Data Availability

The original contributions presented in this study are included in the article. Further inquiries can be directed to the corresponding author.

## Abbreviations

The following abbreviations are used in this manuscript:

3D MOT	Three-dimensional multi-object tracking
RFS	Random finite set
FGO	Factor graph optimization
PMB	Poisson Multi-Bernoulli
TBD	Tracking-by-detection
JDT	Joint detection and tracking
